# Occupational causes of chronic obstructive pulmonary disease: an update

**DOI:** 10.1097/ACI.0000000000000817

**Published:** 2022-02-07

**Authors:** Sara De Matteis

**Affiliations:** Department of Medical Sciences and Public Health, University of Cagliari, Cagliari, Italy

**Keywords:** cleaning products, COPD, occupational lung diseases, occupations, pesticides

## Abstract

**Purpose of review:**

This brief narrative review aims to highlight relevant recent updates on occupational causes of chronic obstructive pulmonary disease (COPD).

**Recent findings:**

The most recent literature has been searched for any new relevant association between occupational exposures and COPD. Only large epidemiological studies of high quality have been included. Beyond the more traditional exposures, such as mineral or organic dusts, new chemicals have emerged as potential occupational causal agents for COPD. In particular, pesticides and cleaning products, including disinfectants, that have shown also positive exposure-response trends. For cleaning products, some specific chemicals have been identified, but for pesticides the identification of specific causal compounds is more challenging. The biological underlying mechanisms are still under study.

**Summary:**

In the recent literature, occupational exposure to pesticides and cleaning products has emerged as potential cause of COPD. Awareness on occupational causes of COPD should increase among all stakeholders, from health professionals to public to prevent the associated public health burden. More studies on identifying the specific causal agents and mechanisms are needed to focus preventive strategies.

## INTRODUCTION

Recently it has been estimated that globally in females and males, most chronic respiratory disease-attributable deaths and disability-adjusted life-years (DALYs) are due to chronic obstructive pulmonary disease (COPD) with rates of 42 deaths per 100 000 individuals (5.7% of total all-cause deaths) and 1068 DALYs (3.3%), respectively [1]. Therefore, it is pivotal to identify all modifiable risk factors to prevent the associated public health burden. Occupational risks have been reported as important preventable causes of COPD, after tobacco smoking, and in some world low-income regions, such as in South-Asia and sub-Saharan Africa, they are estimated as the leading COPD risk factors [1]. It has been previously quantified and recently confirmed [2^▪^] that about 15% of all COPD cases are work-related, but this is likely to be an underestimation of the true occupational COPD burden due to underreporting by healthcare professionals of occupational respiratory diseases (as of all occupational diseases in general), and for lack of a reliable global database on prevalence of workers’ exposure to potential occupational respiratory hazards.

In terms of industries, mining, manufacturing, agriculture, construction, metals, and textile sectors, have been traditionally associated to an increased COPD risk, but an agreed exhaustive list of high-risk COPD jobs does not exist (and maybe it is unachievable). In terms of potential underlying causative agents, the knowledge gap is even bigger; if for some jobs the causal agent is clearer (e.g., coal and silica dusts in mining), in most of epidemiological studies on occupational COPD, only the generic exposure category of ‘vapors, gas, dust or fumes’ (VGDF) is reported, that is of limited value to focus preventive measures [2^▪^]. The real challenge is to identify specific jobs and agents causally associated with COPD risk in old and new work scenarios in order to implement efficient and effective preventive strategies. This is even more challenging in the fast-changing world of work constantly introducing new technologies, but also new potential respiratory hazards at workplace [3].

The aim of this brief narrative review is to highlight the most recent relevant new causes of occupational COPD. 

**Box 1:**
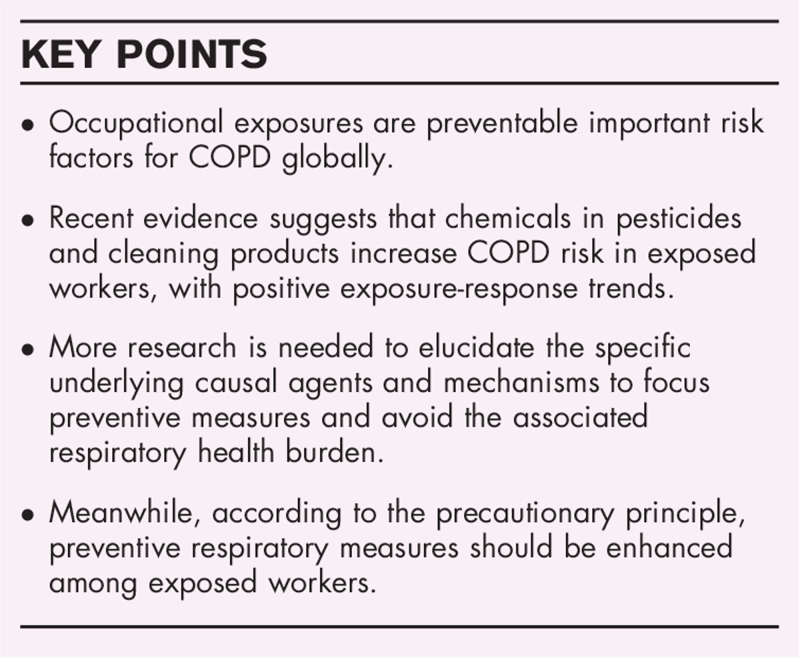
no caption available

## METHODS

A brief literature search on Pubmed database from the last 18 months was performed using MeSH and free text terms related to ‘COPD’ and ‘occupations’, but given that COVID-19 pandemic has dominated the recent research output worldwide, the search was extended to 2019. Inclusion criteria were English language, large epidemiological studies, careful adjustment for tobacco smoking, valid definition of COPD, and reliable occupational exposure assessment.

## RESULTS

In terms of new occupations, an important contribution to the literature comes from a recent large population-based study in the UK Biobank cohort that given the unprecedented size (over half-million subjects) had the opportunity to evaluate complete lifetime job-histories from over 100 000 individuals with spirometry-data and to restrict the analyses to never-smokers and never-asthmatics to rule out any confounding by tobacco smoking, and misclassification with asthma, respectively [4^▪▪^]. Of note, COPD was defined using only acceptable and repeatable individual spirometry data in accordance with the American Thoracic Society/European Respiratory Society guidelines as forced expiratory volume in 1 s (FEV1)/ forced vital capacity (FVC) below the lower limit of normal (i.e., the 5% lower tail of the normal distribution of the average predicted FEV1/FVC ratio in a reference healthy never-smoking population).

**FIGURE 1:**
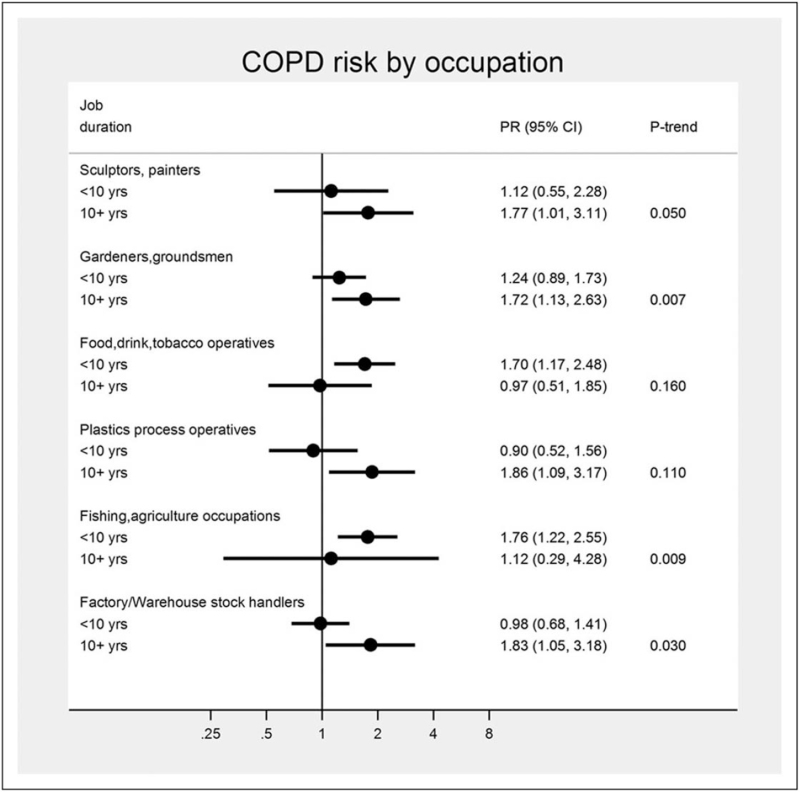
Six occupations at increased chronic obstructive pulmonary disease risk and exposure-response trends by job duration (10 years categories) in the UK Biobank study, 2006–2010, UK. 95% CI, confidence interval; PR, prevalence ratio; *P*-trend, *p*-value for job duration trend test [5^▪▪^].

Six occupations emerged at increased COPD risk in the manufacturing and agriculture sectors: “sculptor, painter, engraver, art restorer”;“gardener, groundsman, park keeper”; “food, drink and tobacco processor”; “plastics processor, moulder”; “agriculture, and fishing occupations not elsewhere classified”; and “warehouse stock handler, stacker.” (Fig. 1).

Of note, significant negative trends by 10-years job duration emerged for agriculture-related jobs, likely due to a *‘*healthy worker survivor effect’ bias (i.e., negative selection of workers with respiratory symptoms from these jobs). Also, new occupations, such as “gardener, groundsman, park keeper” emerged at increased COPD risk, but other previously reported jobs, such as ‘coal miners’ did not, likely due to the underrepresentation of this and other traditional ‘blue collar’ jobs in this voluntary general population cohort of a western high-income country.

**FIGURE 2:**
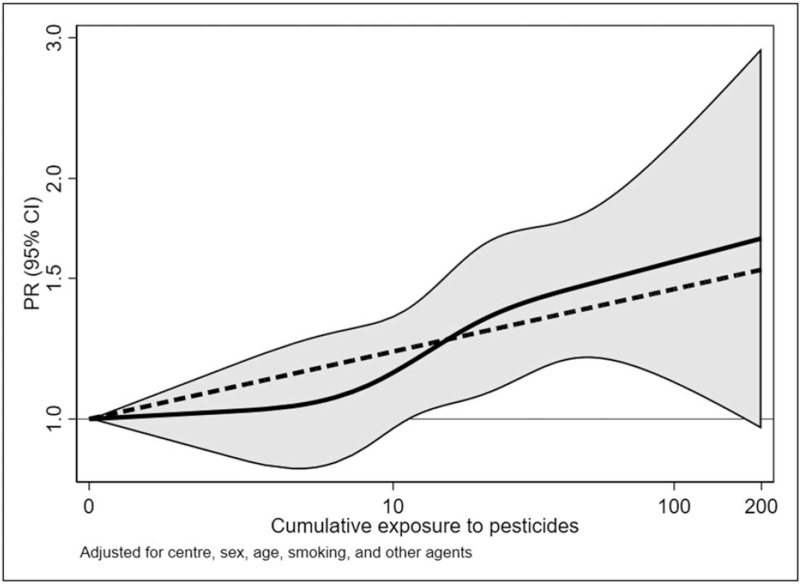
Association between fully adjusted prevalence ratios of chronic obstructive pulmonary disease and cumulative exposure to pesticides (EU-years, ln-transformed) using restricted cubic splines (knots at 25th, 50th, 75th, and 90th percentiles of cumulative exposure among exposed, ln-transformed), in the UK Biobank study, 2006–2010, UK. The continuous curves are prevalence ratios and 95% confidence bands; the dashed line shows the log-linear relationship prevalence ratios  = 1.08 per ln(EU-years). ln, natural logarithm [5^▪▪^].

In relation to new occupational agents, the follow up of the same study [5^▪▪^] applied a general population job-exposure matrix (JEM) called ALOHA+, to evaluate the potential underlying causal agents of the jobs found associated with COPD risk and to find new causal exposures across jobs. The JEM included twelve agents (biological dusts, mineral dusts, gases and fumes, herbicides, insecticides, fungicides, aromatic solvents, chlorinated solvents, other solvents, and metals, all pesticides and vapors/gases/dusts/fumes – VGDF) and assigned, based on expert-assessment, a level of exposure (0 = no, 1 = low, or 2 = high) to each ISCO (International Standard Classification of Occupations)-coded job held in life by study participants (blind to COPD case status) to allow calculation of semi-quantitative cumulative exposure estimates for each agent by multiplying duration of exposure and squared intensity. Beyond the study's strengths already mentioned in the previous job-title analysis, another merit was the adjustment for co-exposure to the JEM agents to try to disentangle the specific agent effects. Pesticides’ exposure showed increased COPD risks for ever exposure (prevalence ratio -PR = 1.13;95%confidence interval-CI:1.01–1.28), and for high cumulative exposure (prevalence ratio = 1.32;95%CI:1.12–1.56), with positive exposure-response trends (*P*-trend = 0.004) (Fig. 2).

This finding was previously reported by a few smaller studies [6,7], and so this result is important to support the validity of this association, still highly debated [8^▪^,^9^].

Another relevant recent study on new occupational exposures for COPD risk was conducted in the Nurses’ Health Study II, a large US prospective cohort of female nurses, to investigate the association between exposure to cleaning products and disinfectants and COPD incidence [10^▪▪^]. In about 70 000 nurses, occupational exposure to cleaning products and disinfectants was evaluated by questionnaire and a job-task exposure matrix (JTEM). The JTEM assigned an exposure level (low, medium, or high) based on types of nursing jobs and general disinfection tasks, and allowed to evaluate exposure to specific cleaning agents and disinfectants. Several potential confounders, in particular tobacco smoking, and ethnicity, were taken into account. Of note, COPD was based only on a self-reported doctor's diagnosis, and not on spirometry data.

**FIGURE 3:**
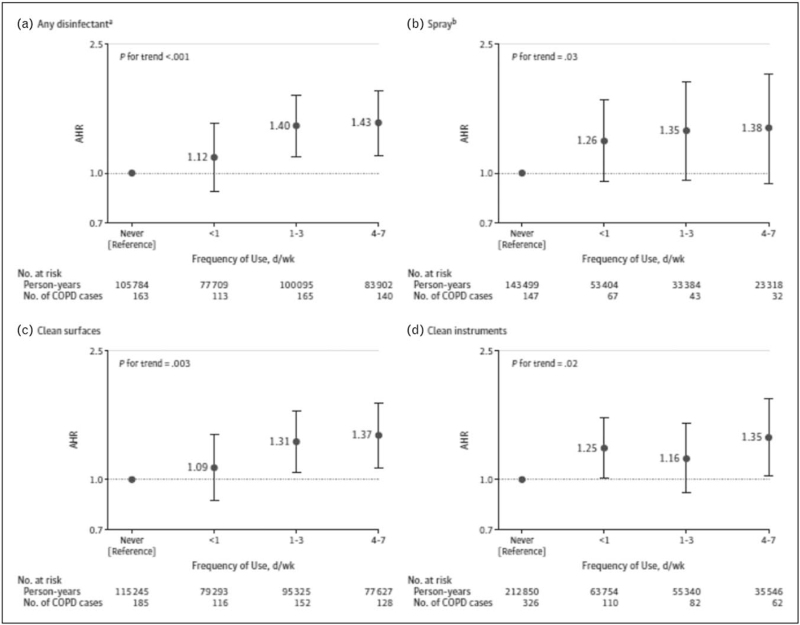
Prospective association between self-reported frequency of cleaning/disinfection tasks and chronic obstructive pulmonary disease incidence among us female nurses. Occupational exposure was evaluated as the highest exposure level at any of the questionnaire cycles before time of diagnosis. The follow-up periods were 2009--2015 for job type and use of disinfectants and 2011--2015 for use of sprays. Multivariable models were adjusted for age, smoking status and pack-years (continuous), race, ethnicity, and body mass index. Observations with missing values for pack-years of smoking (<0.5%) were excluded from analyses. Observations with missing values for body mass index (3.8%) were included in the model as a ‘missing’ category. Error bars indicate 95% confidence intervals (CIs). Adjusted hazard ratios with 95% CIs are shown for type of disinfectant (A and B) and specific use of disinfectant (C and D). a Use of a disinfectant to clean surfaces or instruments. b Use of sprays for patient care, instrument cleaning or disinfection, surface cleaning or disinfection, air refreshing, or other [10^▪▪^].

The authors found that weekly use of disinfectants to clean surfaces and to clean medical instruments was associated with increased COPD incidence, with adjusted hazard ratios of 1.38 (95% CI, 1.13–1.68) and 1.31 (95% CI, 1.07–1.61), respectively. High-level exposure, evaluated by the JTEM, to several specific disinfectants (i.e., glutaraldehyde, bleach, hydrogen peroxide, alcohol, and quaternary ammonium compounds) was associated with COPD incidence, with adjusted hazard ratios ranging from 1.25 (95% CI, 1.04–1.51) to 1.36 (95% CI, 1.13–1.64). (Fig. 3).

**FIGURE 4:**
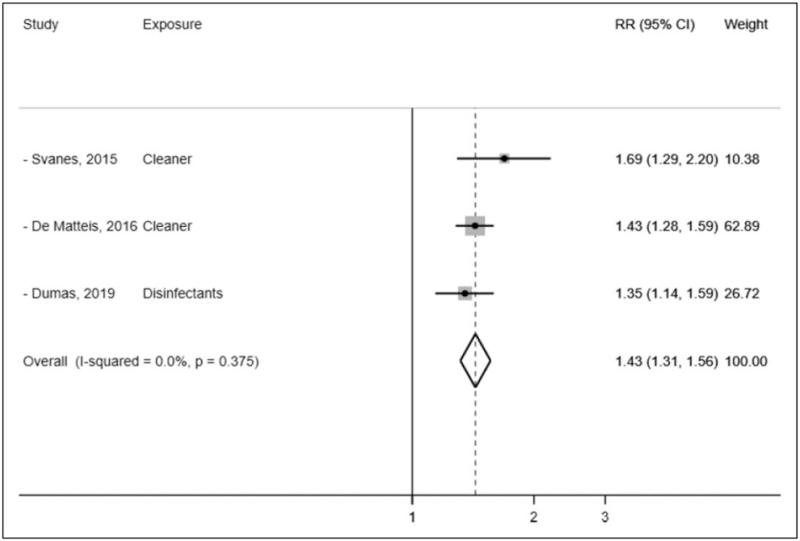
Meta-analysis of three studies evaluating the association between occupational cleaning exposure and COPD risk. COPD, chronic obstructive pulmonary disease; RR, relative risk [12^▪^].

The association in this large longitudinal prospective study adds to the evidence supporting the debated hypothesis that chronic exposure to irritants, such as cleaning products and disinfectants may cause COPD [11^▪^]. Of note, this study has informed a recent meta-analysis, that has quantified in 43% the increased COPD risk for occupational exposure to cleaning products (Fig. 4) [12^▪^].

## DISCUSSION

In the most recent literature, besides more traditional occupational exposures (e.g., mineral and organic dusts) associated with increased COPD risk, emerging causal agents are chemicals in pesticides, and cleaning products, including disinfectants.

Occupational exposure to both agents have been previously associated to respiratory health effects, in particular asthma risk [8^▪^,11^▪^,12^▪^], so the causal link with COPD is plausible, considering that both are chronic respiratory diseases, characterized by underlying airway inflammation and so potentially sharing similar etiopathogenetic pathways. The underlying biological mechanisms are still under study. For cleaning agents both sensitiser- and irritant-induced respiratory health effects have been reported in experimental studies [11^▪^]. In particular, an immunoglobulin E-mediated mechanism has been suggested for few compounds, such as chloramine-T, ortho-phthalaldehyde, and enzymes. However, in the majority of studies, irritation has been reported as a potential causal mechanism. In particular, sensory irritation (via interaction of a chemical agent with receptors of the nervous system) can occur, such as in the afferent nerve endings in the airways, where two key transient receptor potential (TRP) channels are present: TRPV1 and TRPA1. TRPV1 is activated by capsaicin, but also by low pH, ethanol, or eugenol. TRPA1 is considered a major irritant detector and is activated by a broad range of irritants, such as formaldehyde, hydrogen peroxide, hypochlorite. Upon activation of the sensory nerves, various neuropeptides, such as substance P, neurokinin A, and calcitonin-gene-related peptide are released locally. These neuropeptides trigger an airway neurogenic inflammation which reflects the transition from pure sensory, reversible effects to general and inflammatory defence mechanisms.

Also, tissue irritation has been reported, that is characterized by direct epithelial damage of the airways induced by an irritant agent. When physiological repair capacities are overwhelmed, initial adaptive responses can be followed by inflammation and irreversible tissue damage.

Several sensitizers also have irritant properties, including disinfectants (glutaraldehyde, quaternary ammonium salts, chloramine-T, isothiazolinone), ethanolamine and enzymes, so both mechanisms can act synergically [11^▪^].

In relation to pesticides, exposure to cholinesterase (ChE) inhibiting pesticides has been associated with a decreased FEV1/FVC [8^▪^]. In relation to biological plausibility, ChE-inhibiting pesticides such as organophosphate have cholinergic effects resulting in increased bronchial secretion and bronchoconstriction. Also, neutrophilic and oxidative stress-mediated inflammation have been hypothesized for pesticide-related chronic respiratory diseases pathogenesis and a recent mechanistic study found that stimulation of alveolar macrophages and increase of NF-kB activation, resulting in TNF-α protein release, could be an additional underlying biological mechanism [13].

Exposure to both pesticides and cleaning products can occur beyond the workplace as para-occupational (e.g., for cohabitants of pesticides’ appliers) or environmental (e.g., among children due to domestic use of cleaning products), so the respiratory public health burden could be higher than just the occupational one. Of note, in the current COVID-19 pandemic, the use of disinfectants has become ubiquitous so to better understand the respiratory health effects of these agents is a key public health issue.

As, for other occupational causes of respiratory diseases, an integrated multidisciplinary approach involving all stakeholders is needed to prevent or at least reduce the associated public heath burden:

(1) Worldwide governments should make sure that occupational health and safety regulatory bodies establish and enforce evidence-based occupational exposure limits for respiratory hazards, and regularly re-evaluate protections currently in place based on new relevant evidence.
(2) Employers must provide their workers not only appropriate personal protective equipment (e.g., high protection face masks), but also, most importantly, access to an occupational health service to ensure health and safety information and training, and periodic respiratory health surveillance for early detection of any early adverse health effect.
(3) To increase clinical recognition of occupational COPD and in general of all occupational respiratory diseases, the core training and subsequent continuing medical education of health professionals should include occupational medicine.
(4) Given the fast technological progress, new agents are constantly introduced at workplaces that can have potential harm on respiratory system. Presence at workplace of potential respiratory irritants, labelled with the hazard statement H335 (’May cause respiratory irritation’) according to the Globally Harmonized System of Classification and Labelling of Chemicals, should trigger implementation of preventive measures according to the precautionary principle.
(5) Further epidemiological studies, especially in low-income countries (often overlooked, but likely at higher risk), are needed. Ideally prospective large cohorts using more precise quantitative exposure assessment of individual agents (e.g., exact chemical composition by use of product bar codes), detailed clinical phenotyping (e.g., airway inflammatory and immune biomarkers) and modern molecular methods (e.g., -omics) would help clarify both the underlying causal agents and the relevant biological mechanisms. To achieve this goal, international research funding schemes should increase their support to occupational respiratory research, currently regrettably underfunded.

## CONCLUSION

This review supports the hypothesis that occupational exposure to both pesticides and cleaning products may be new causes of COPD. Hazard identification is the first step for prevention, so more research is needed, globally, to elucidate the specific underlying agents and mechanisms involved. Filling this knowledge gap would allow implementation of effective focused preventive intervention strategies aimed to eliminate or at least control exposure to these hazardous chemicals and identify early health effects to prevent the associated occupational respiratory health burden with important personal, medical, and societal benefits.

## Acknowledgements

*None.*

### Financial support and sponsorship

*None.*

### Conflicts of interest

*There are no conflicts of interest.*

## REFERENCES AND RECOMMENDED READING

Papers of particular interest, published within the annual period of review, have been highlighted as:

▪ of special interest
▪▪ of outstanding interest
